# C-Reactive Protein Promotes Diabetic Kidney Disease in db/db Mice via the CD32b-Smad3-mTOR signaling Pathway

**DOI:** 10.1038/srep26740

**Published:** 2016-05-25

**Authors:** Yong-Ke You, Xiao-Ru Huang, Hai-Yong Chen, Xia-Fei Lyu, Hua-Feng Liu, Hui Y. Lan

**Affiliations:** 1 Institute of Nephrology, Guangdong Medical College, Zhanjiang, Guangdong, China; 2 Department of Medicine and Therapeutics, and Li Ka Shing Institute of Health Sciences, and Shenzhen Research Institute, the Chinese University of Hong Kong, Hong Kong, China

## Abstract

C-reactive protein (CRP) is associated with progressive diabetic nephropathy in patients with type-2 diabetes (T2DN). However, role of CRP in T2DN remains unclear. We report here that CRP is pathogenic in T2DN in db/db mice that express human CRP (CRPtg-db/db). Compared to the littermate db/db mice, CRPtg-db/db developed more severe T2DN, showing higher levels of fasting blood glucose and microalbuminuria and more progressive renal inflammation and fibrosis. Enhanced T2DN in CRPtg-db/db mice were associated with over-activation of CRP-CD32b, NF-κB, TGF-β/Smad3, and mTOR signaling. Further studies *in vitro* defined that CRP activated Smad3 directly at 15 mins via the CD32b- ERK/p38 MAP kinase crosstalk pathway and indirectly at 24 hours through a TGF-β1-dependent mechanism. Importantly, CRP also activated mTOR signaling at 30 mins via a Smad3-dependent mechanism as Smad3 bound mTOR physically and CRP-induced mTOR signaling was abolished by a neutralizing CD32b antibody and a specific Smad3 inhibitor. Finally, we also found that CRP induced renal fibrosis through a CD32b-Smad3-mTOR pathway because blocking mTOR signaling with rapamycin inhibited CRP-induced CTGF and collagen I expression. Thus, CRP is pathogenic in T2DN. CRP may promote CD32b- NF-κB signaling to mediate renal inflammation; whereas, CRP may enhance renal fibrosis in T2DN via CD32b-Smad3-mTOR signaling.

Diabetes mellitus (DM) has become a major global health problem with high morbidity and mortality. Type 2 diabetic nephropathy (T2DN) is one of the most important long-term microvascular complications of DM and becomes a leading cause of end-stage renal disease (ESRD) worldwide. Increasing evidence shows that T2DM is a low-grade inflammatory disease^1^. In patients with T2DM, serum levels of pro-inflammatory cytokines such as interleukin-1 beta (IL-1β), interleukin-6 (IL-6), and CRP (C-reactive protein) are elevated and have been widely used as a biomarker of T2DM^1^,^2^,^3^,^4^. This is particularly important in those with DN^5^,^6^, suggesting a close relationship between inflammation and T2DM/T2DN.

CRP is an acute-phase protein and is rapidly synthesized and released in response to inflammation and tissue damage^7^. In patients with T2DM, elevated serum levels of CRP are closely associated with an increase in microalbuminuria and renal dysfunction^4^,^5^, suggesting the close link between CRP and the development of DN. Among the inflammatory cascade, CRP can induce IL-6 via a NF-κB-dependent mechanism^8^. We also found that under diabetic conditions, CRP is induced by high glucose, which in turn synergistically promotes high glucose-mediated renal inflammation and fibrosis *in vitro* and in a mouse model of streptozotocin-induced type-1 diabetes^9^. The functional importance for CRP is also demonstrated in other disease models including obstructive nephropathy^10^, ischemic kidney injury^11^, hypertensive heart disease^12^, and atherosclerosis^13^. However, the pathogenic role and regulatory mechanisms of CRP in T2DN remain unclear. Thus, the present study examined the pathogenic importance of CRP on T2DN by transgenically overexpressing human CRP in db/db mice. The mechanism whereby CRP promoted renal fibrosis through the CD32b-Smad3-mTOR mechanism was identified *in vivo* and *in vitro*.

## Results

### CRP enhances fasting blood glucose and diabetic kidney injury in CRPtg-db/db mice

**Figure 1:**
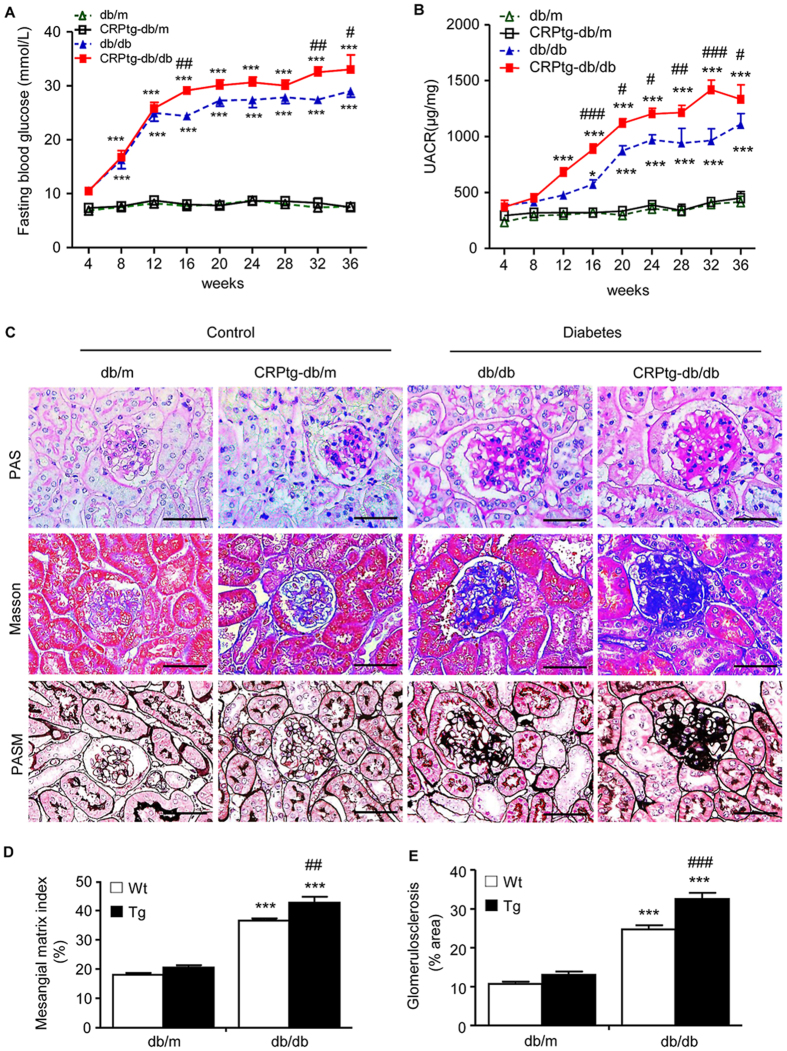
CRPtg-db/db mice develop more severe diabetic renal functional and histological injury. (**A**) Fasting blood glucose. (**B**) Urinary albumin to creatinine ratio (UACR). (**C**) Periodic acid Schiff (PAS), Masson’ trichrome and periodic acid-silver methenamine (PASM) staining. (**D,E**) Quantitative analysis of mesangial expansion and glomerulosclerosis. Data represents the mean ± SEM for eight mice per group. Bar = 50 μm. *p < 0.05, **p < 0.01, ***p < 0.001 compared with db/m mice; ^#^*p* < 0.05, ^##^*p* < 0.01, ^###^*p* < 0.001 compared with db/db mice.

An equal high level of fasting blood glucose was demonstrated in both db/db and CRPtg-db/db mice at the age of week 4; however, CRPtg-db/db mice developed higher levels of fasting blood glucose at week 16 onwards (Fig. 1A). This was associated with the development of the significant higher levels of microalbuminuria as determined by the urinary Albumin-to-Creatinine Ratio (UACR) in CRPtg-db/db mice over 16–36 weeks (Fig. 1B). However, compared to db/db mice, levels of serum creatinine and blood urea nitrogen were only marginally increased in CRPtg-db/db mice (Supplementary Fig. S1). Histologically, periodic acid–Schiff (PAS), Masson trichrome, and Periodic Schiff-Methenamine Silver (PASM) staining revealed that db/db mice developed moderate mesangial matrix deposition, thickening of glomerular basement membrane, tubulointerstitial extracellular matrix accumulation, and glomerulosclerosis when compared to the littermate db/m and CRPtg-db/m (Fig. 1C–E). All these pathological changes became much more severe in CRPtg-db/db mice (Fig. 1C–E).

### Overexpression of CRP in db/db mice largely promotes renal inflammation and fibrosis

**Figure 2:**
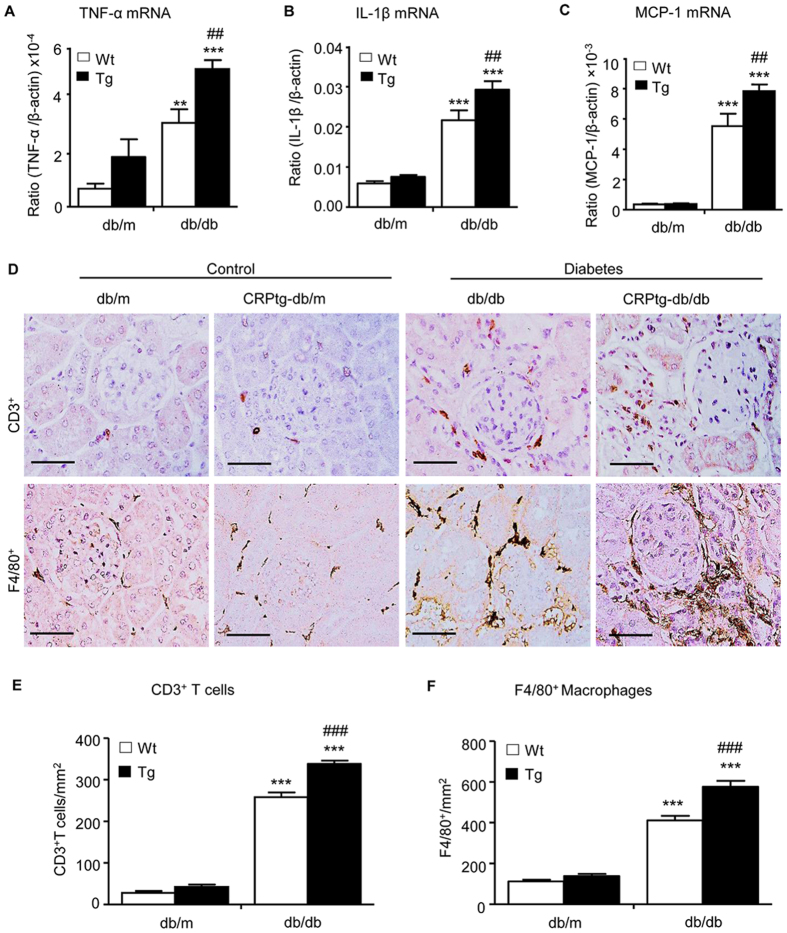
Renal inflammation is enhanced in CRPtg-db/db mice. (**A–C**) Real-time PCR analysis of pro-inflammatory cytokines of TNF-α, IL-1β, MCP-1, respectively. (**D–F**) Immunohistochemical staining and quantitative analysis of infiltration of CD3^+^ T cells and F4/80^+^ macrophage cells. Data represents the mean ± SEM for eight mice per group. Bar = 50 μm. **p < 0.01, ***p < 0.001 compared with db/m mice; ^##^p < 0.01, ^###^p < 0.001 compared with db/db mice.

**Figure 3:**
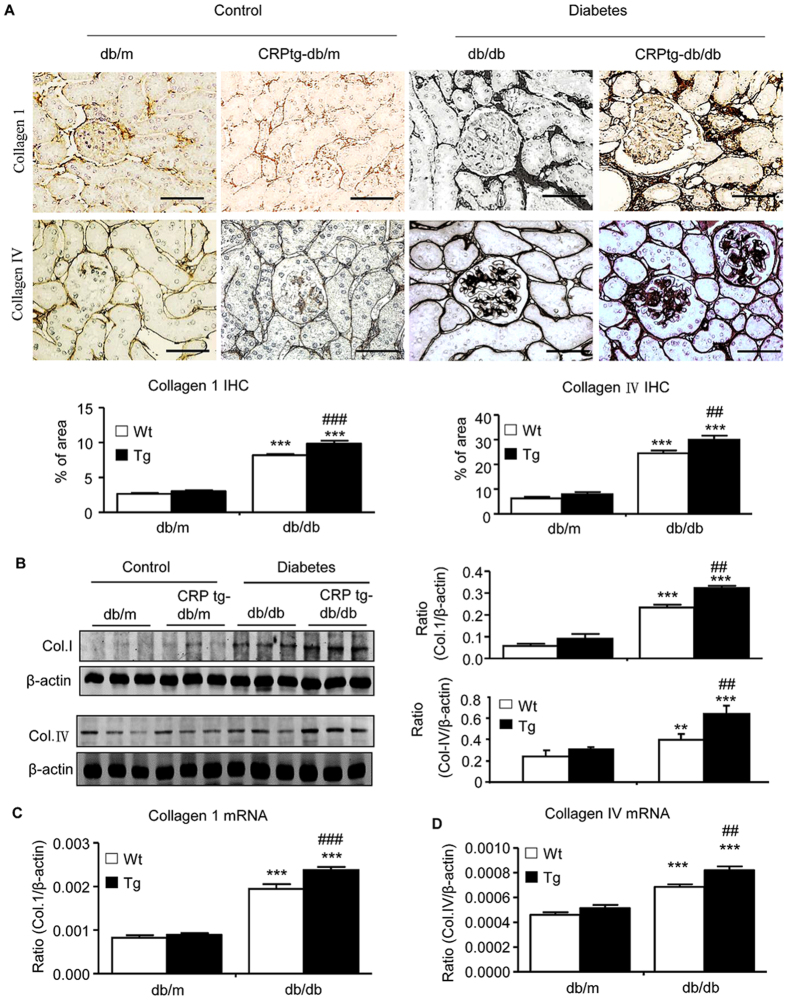
Renal fibrosis is enhanced in CRPtg-db/db mice. (**A**) Collagen I and collagen IV expression examined by immunohistochemistry. (**B**) Collagen I and collagen IV expression examined by Western blotting analysis. (**C,D**) Real-time PCR analysis of collagen 1 mRNA and collagen IV mRNA expression, respectively. Data represents the mean ± SEM for eight mice per group. Bar = 50 μm. **p < 0.01, ***p < 0.001 compared with db/m mice; ^##^*p* < 0.01, ^###^*p* < 0.001 compared with db/db mice.

We then examined whether CRP-enhanced renal functional injury in db/db mice is associated with more severe renal inflammation and fibrosis, two major pathological features related to the development of DN. Immunohistochemistry and real-time PCR analysis revealed that compared with the littermates db/m and CRPtg-db/m control mice, db/db mice devolved moderate renal inflammation including a marked up-regulation of pro-inflammatory cytokines/chemokines (TNF-α, IL-1β, and MCP-1) and renal infiltration of F4/80^+^ macrophages and CD3^+^ T cells at the age of week 36 (Fig. 2 and Supplementary Fig. S2). All of these inflammatory features in the diabetic kidney of db/db mice were largely exacerbated in CRPtg-db/db mice (Fig. 2 and Supplementary Fig. S2). Further studies also revealed that moderate renal fibrosis such as expression of collagen I and IV mRNA and their matrix protein accumulation in db/db mice at week 36 was also largely enhanced in CRPtg-db/db mice (Fig. 3).

### Enhanced renal inflammation and fibrosis in CRPtg-db/db mice are associated with the activation of CD32b-NF-κB and CD32b-Smad3-mTOR signaling pathways

**Figure 4:**
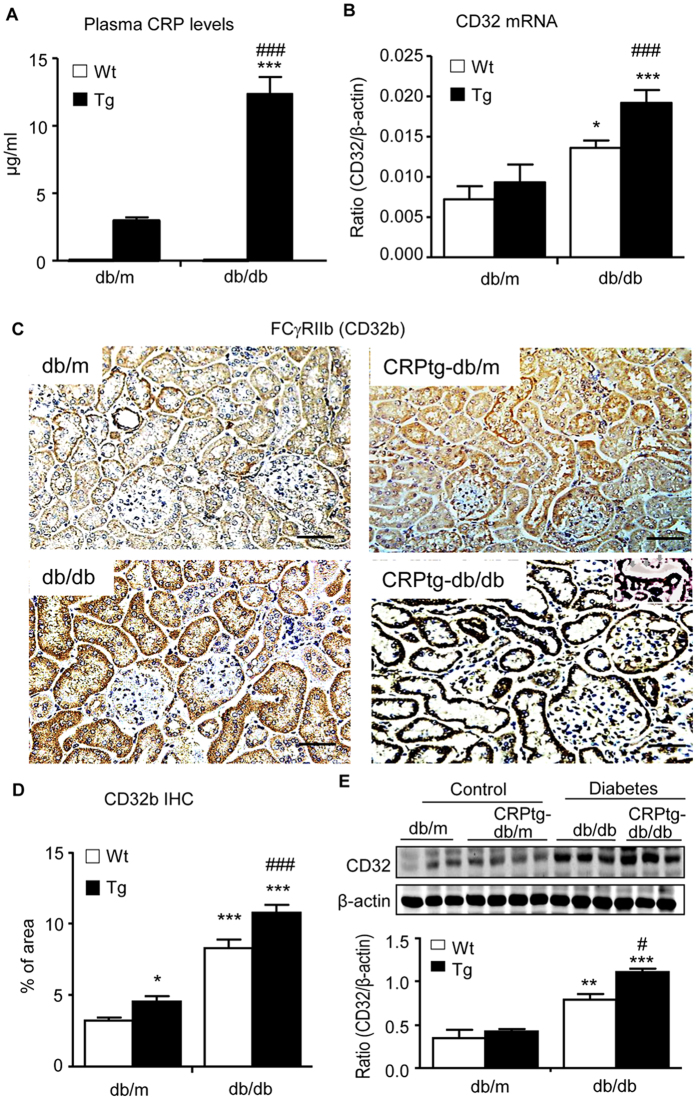
Expression of Fc gamma Receptor IIB (CD32b) is largely enhanced in the diabetic kidney of CRPtg-db/db mice. (**A**) Plasma levels of human CRP. (**B**) CD32b mRNA expression by real-time PCR. (**C**) CD32b expression pattern detected by immunohistochemical staining. Note that CD32b is highly expressed by tubular epithelial cells and podocytes (inserted picture) in CRPtg-db/db mice. (**D**) Quantitative analysis of CD32b expression. (**E**) Western blot analysis. Data represents the mean ± SEM for eight mice per group. Bar = 50 μm. *p < 0.05, **p < 0.01, ***p < 0.001 compared with db/m mice; ^#^*p* < 0.05, ^###^*p* < 0.001 compared with db/db mice.

**Figure 5:**
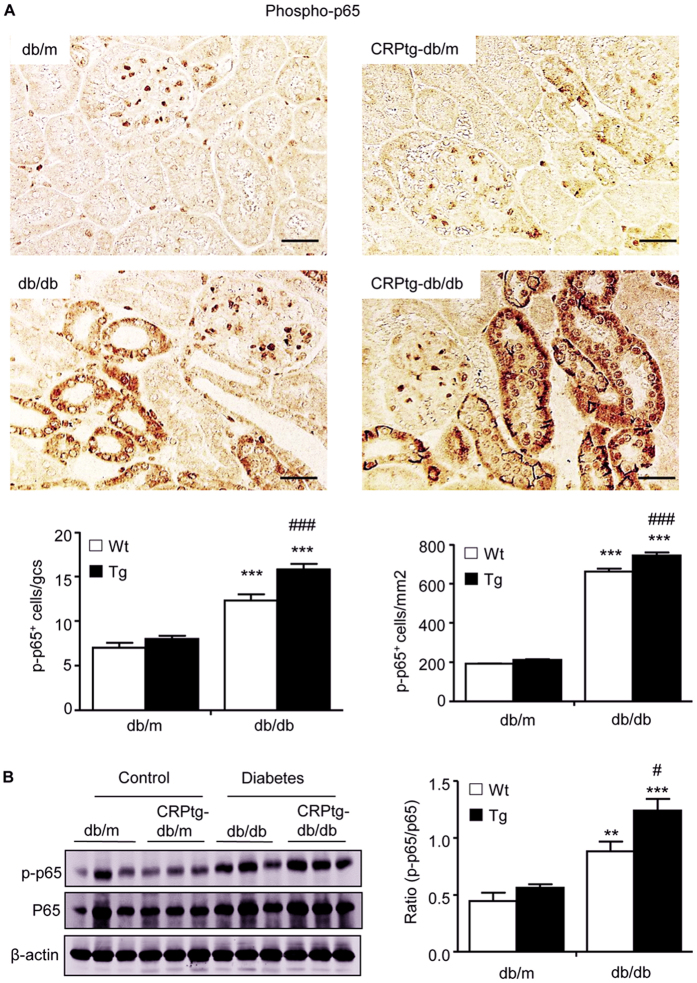
NF-κB signaling is significantly increased in CRPtg-db/db mice. (**A**) Immunohistochemical staining and quantitative analysis of nuclear phospho-NF-κB/p65 (p-p65). (**B**) Western blot and quantitative analysis of phospho-NF-κB/p65 (p-p65) protein. Data represents the mean ± SEM for eight mice per group. Bar = 50 μm. **p < 0.01, ***p < 0.001 compared with db/m mice; ^#^p < 0.05, ^###^p < 0.001 compared with db/db mice.

We next investigated the underlying signaling mechanisms by which CRP promotes diabetic kidney injury. Firstly, we examined plasma levels of CRP and its receptor, C32b. As shown in (Fig. 4), compared with db/m mice in which human CRP was undetectable, plasma levels of human CRP were moderate increased in CRPtg-db/m mice, which was largely elevated in CRPtg-db/db mice (Fig. 4A). Real-time PCR, immunohistochemistry, and western blot analysis revealed a significant upregulation of CD32b in db/db, which was further increased in CRPtg-db/db mice. Expression of CD32b was primarily found in tubular epithelial cells and podocytes (Fig. 4C). Further study revealed that upregulation of CD32b in the diabetic kidney of db/db mice was associated with higher levels of phosphorylated p65 subunit and its nuclear translocation, which was further enhanced in CRPtg-db/db mice (Fig. 5), suggesting that CRP may promote renal inflammation in T2DN via a CD32b-NF-κB signaling mechanism.

**Figure 6:**
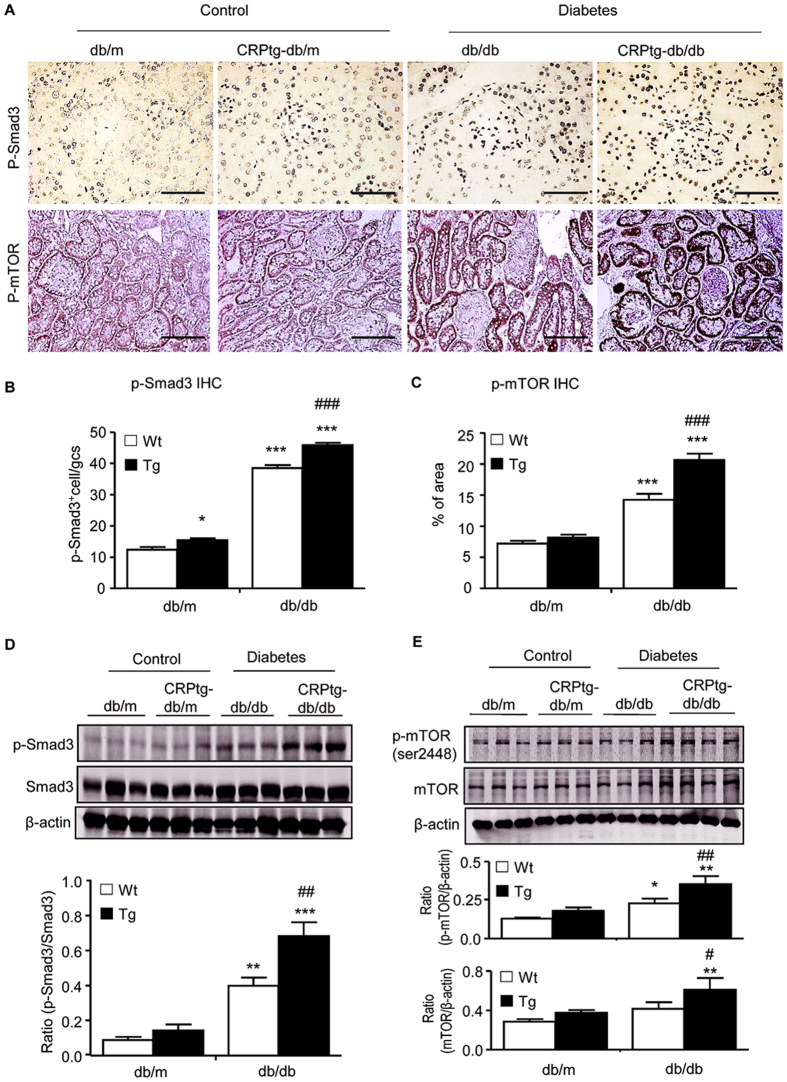
Smad3 and mTOR signaling is largely enhanced in the kidney of CRPtg-db/db mice. (**A–C**) Immunohistochemical staining of phospho-Smad3 and phospho-mTOR, respectively. (**D,E**) Western blot analysis of phospho-Smad3 and phospho-mTOR in the diabetic kidney, respectively. Data represents the mean ± SEM for eight mice per group. Bar = 50 μm. *p < 0.05, **p < 0.01, ***p < 0.001 compared with db/m mice; ^#^*p* < 0.05, ^##^*p* < 0.01, ^###^*p* < 0.001 compared with db/db mice.

Interestingly, we also found that upregulation of CD32b in CRPtg-db/db mice was associated with a significant upregulation of renal TGF-β1 and CTGF at mRNA and protein levels (Supplementary Fig. S3), which was associated with enhanced Smad3 signaling as detected by higher levels of phosphorylated Smad3 and its nuclear localization in glomerular and tubulointerstitial cells (Fig. 6).

Because activation of mTOR has been shown to play an important role in the progression of DN in which TGF-β/Smad3 signaling is highly activated^14^,^15^,^16^, we examined the activation of mTOR signaling in the diabetic kidney by immunohistochemistry and western analysis. We found that like Smad3 signaling, phosphorylated mTOR was largely increased in the diabetic kidney of db/db mice, particularly in tubular epithelial cells and podocytes, which became much more profound in CRPtg-db/db mice (Fig. 6). All these findings suggested a close link of CD32b-Smad3-mTOR signaling in CRP-mediated T2DN.

### CRP activates Smad3 via the CD32b-ERK/p-38 MAP kinase crosstalk pathway *in vitro*

**Figure 7:**
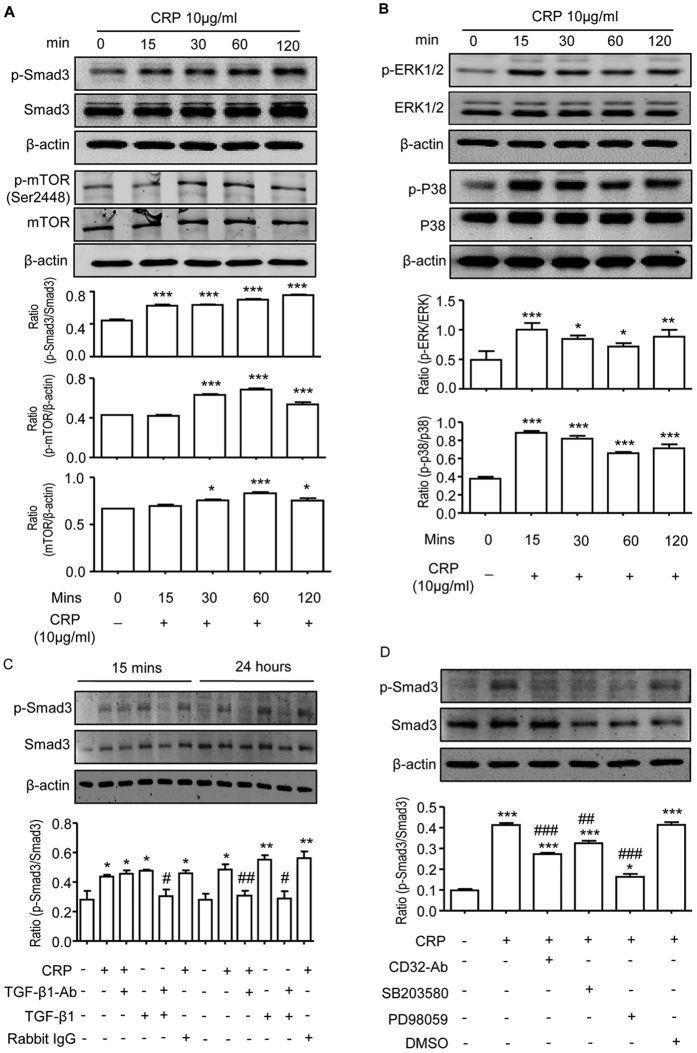
CRP induces activation of Smad3 directly via the CD32b-ERK/p38 MAP kinase-crosstalk pathway and indirectly through the TGF-β1-dependent mechanism in HK-2 cells. (**A**) p-Smad3 and p-mTOR, respectively. (**B**) p-ERK1/2 and p-p38, respectively. Data represents the mean ± SEM for at least three independent experiments. *p < 0.05, **p < 0.01, ***p < 0.001 compared with time 0. (**C**) HK-2 cells were pretreated with anti-TGF-β1 neutralizing antibody (10 μg/ml) or isotype control rabbit IgG (10 μg/ml) for 1 hour before stimulated with CRP (10 μg/ml) for 15 minutes or 24 hours and examined for phosphorylation of Smad3 by western blots. Representative western blots and quantitation of p-Smad3 are shown. (**D**) HK-2 cells were pretreated with anti-CD32b neutralizing antibody (5 μg/ml), and specific inhibitors to ERK1/2 (PD98059, 20 μM), to p38 (SB203580, 10 μM) for 1 hour before stimulated with CRP (10 μg/ml) for 15 minutes. The phosphorylation of Smad3 was detected by western blot analysis. Data represents the mean ± SEM for at least three independent experiments. *p < 0.05, **p < 0.01, ***p < 0.001 compared with normal group; ^#^*p* < 0.05, ^##^*p* < 0.01, ^###^*p* < 0.001 compared with addition of CRP.

Because TGF-β/Smad3 signaling was highly activated in the diabetic kidney associated with a marked upregulation of CD32b and severe renal fibrosis in CRPtg-db/db mice (Figs 3, 4, 6 and Supplementary Fig. S3), we hypothesized that CRP may activate Smad3 via the CD32b-dependent mechanism, which was investigated *in vitro* in HK-2 tubular epithelial cells. We found that addition of CRP (10 μg/ml) was able to induce Smad3 phosphorylation in a time-dependent manner, being significant as early as 15 mins (Fig. 7A), which was accompanied by a late response at 24 hours (Fig. 7C). Interestingly, CRP-induced Smad3 phosphorylation at 15 mins was associated with activation of the ERK1/2 and p38 (Fig. 7B), suggesting a link between ERK/p38 and Smad3 signaling. This was examined by treating CRP-stimulated HK-2 cells with ERK and p38 inhibitors. As shown in Fig. 7(D), addition of a neutralizing antibody to CD32b or inhibitors to ERK1/2 (PD98059) or p38 (SB203580) was capable of blocking CRP-induced Smad3 phosphorylation at 15 mins, revealing the CD32b-ERK/p38 MAP kinase crosstalk pathway in the early activation of Smad3 signaling in response to CRP. This was further confirmed by the inability of a neutralizing anti-TGF-β1 antibody to block CRP-induced Smad3 phosphorylation at 15 mins, but not at 24 hours (Fig. 7C). Thus, CRP activated the early Smad3 signaling at 15 mins via the ERK/p38 MAP kinase crosstalk pathway and the late Smad3 activation at 24 hours though a TGF-β1-dependent mechanism.

### CRP activates mTOR via a CD32b-Smad3-dependent mechanism *in vitro*

**Figure 8:**
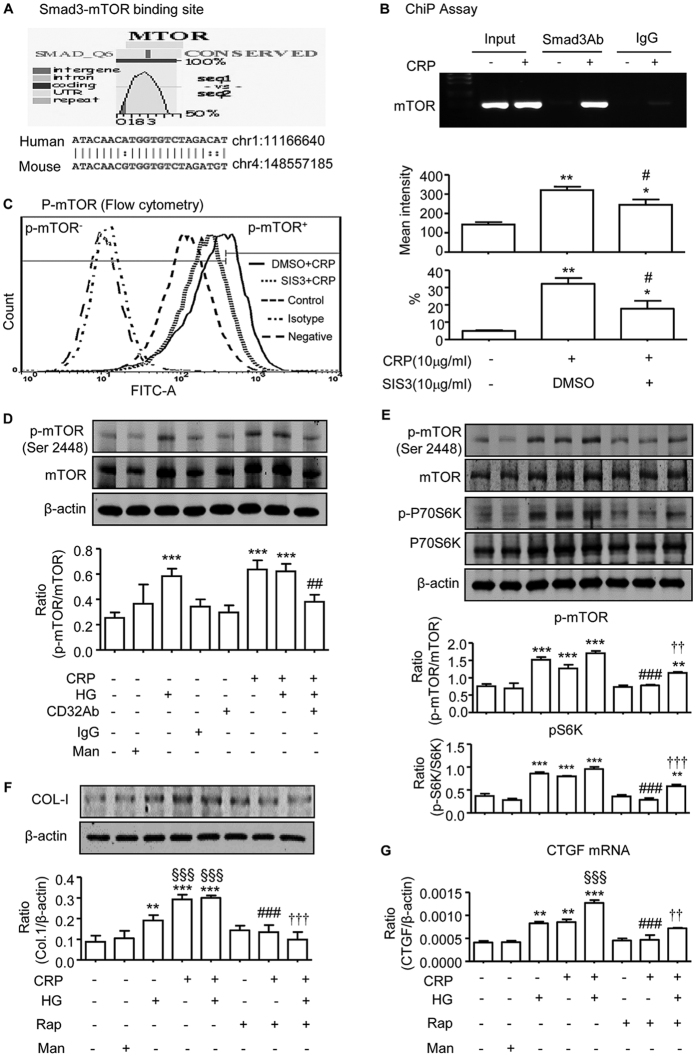
CRP induced Smad3 and mTOR signaling was blocked by a neutralizing CD32b antibody and a specific Smad3 inhibitor, and blockade of mTOR signaling with rapamycin inhibited CRP-induced collagen I expression. (**A**) Smad3-mTOR binding site. (**B**) ChIP assay detects the physical interaction between Smad3 and mTOR, which is enhanced by addition of CRP (10 μg/ml) in HK-2 cells. (**C**) HK-2 cells were pretreated with SIS3 (10 μM) or DMSO for 1 hour before stimulated with CRP (10 μg/ml) for 1 hour and examined for phosphorylation of mTOR by flow cytometry. Representative flow cytometry histograms and quantitative analysis of mean FITC intensity are shown. Data represents the mean ± SEM for at least three independent experiments. *p < 0.05, **p < 0.01 compared with normal group; ^#^p < 0.05 compared with addition of CRP. CRP and high glucose-induced activation of mTOR-p70S6K signaling and collagen I expression in HK-2 cells is blocked by a neutralizing CD32b antibody and rapamycin. (**D**) Representative western blots show that addition of CRP (10 μg/ml) or high glucose (35 mM) induces phosphorylation of mTOR, which is blocked by a neutralizing anti-CD32b antibody (5 μg/ml). (**E**) Representative western blots show that addition of CRP (10 μg/ml) or high glucose (35 mM) induces phosphorylation of mTOR and S6K, which is blocked by rapamycin (10 μM). (**F,G**) Blocking mTOR signaling with rapamycin is able to inhibit CRP and/or high glucose-induced upregulation of collagen I (**F**) and CTGF expression (**G**). Data represents the mean ± SEM for at least three independent experiments. **p < 0.01, ***p < 0.001 compared with normal group; ^##^*p* < 0.01, ^###^*p* < 0.001 compared with CRP group; ^§§§^*p* < 0.001 compared with HG group; ^††^*p* < 0.01, ^†††^*p* < 0.001 compared with CRP + HG group.

We next investigated signaling mechanism whereby CRP activates mTOR in HK-2 cells. By using an ECR browser^17^, we found a Smad3 binding site on the UTR of mTOR gene (Fig. 8A). A ChIP assay also clearly detected the physical interaction of Smad3 with the UTR region of mTOR as identified by the ability of an antibody against Smad3 to successfully immunoprecipitate the DNA fragments that contain the potential Smad3 binding site in the UTR regions of mTOR, which was largely enhanced by CRP (Fig. 8B). The functional link between Smad3 and mTOR signaling was further demonstrated by the findings with quantitative flow cytometry, immunofluorescence, and western blot analysis that blockade of Smad3 signaling with a specific inhibitor (SIS3) was able to inhibit CRP-induced mTOR signaling in HK-2 cells in a dose-dependent manner (Fig. 8C, and Supplementary Figs S4 and S5), revealing a Smad3-mTOR signaling in response to CRP.

We have previously shown that high glucose is able to induce CRP expression by tubular epithelial cells^9^. Thus, we investigated the effect of CRP on activation of mTOR signaling under high glucose conditions. As shown in (Fig. 8D), addition of CRP (10 μg/ml) or high glucose (35 mM), but not control mannitol (35 mM), was capable of activating mTOR as evidenced by a large increase in phosphorylated mTOR protein. Interestingly, the combination of CRP and high glucose did not additively increase mTOR signaling. However, addition of a neutralizing antibody to CD32b blunted both high glucose and CRP-induced mTOR signaling (Fig. 8D), implying that high glucose induces mTOR signaling via the CRP-CD32b-dependent mechanism. We also found that addition of CRP (10 μg/ml) and/or high glucose (35 mM) for 6 hours could activate both mTOR and its substrate p70-S6 kinase, which was blocked by rapamycin (10 μM), an mTOR inhibitor (Fig. 8E). Furthermore, blockade of mTOR signaling with rapamycin was also able to inhibit CRP and /or high glucose-induced upregulation of collagen I and CTGF expression (Fig. 8F,G), demonstrating mTOR signaling as a central mechanism by which CRP promotes diabetic kidney disease in T2DN.

## Discussion

We report here an important role of human CRP in Type 2 diabetic kidney disease as CRPtg-db/db mice developed much more progressive kidney injury including higher levels of hyperglycemia, microalbuminuria, and excessive renal inflammation and fibrosis.

We found that CRP promoted renal inflammation via the CD32b-NF-κB-dependent mechanism, resulting in upregulation of TNF-α, IL-1β and MCP-1, and an increase in CD3^+^ T cell and F4/80^+^ macrophage infiltration in the diabetic kidney of CRPtg-db/db mice, revealing a role for CD32b-NF-κB signaling in CRP-mediated renal inflammation. This finding was consistent with a known mechanism of NF-κB in diabetic renal inflammation^9^,^18^,^19^,^20^,^21^,^22^,^23^,^24^. Indeed, we have previously reported that human CRP can promote renal inflammation by activating NF-κB signaling via the CD32/64-dependent mechanism in a mouse model of type 1 diabetic nephropathy induced in CRPtg mice and *in vitro*^9^. Again, the present study confirmed the CD32b-NF-κB-dependent mechanism in renal inflammation in T2DN induced in CRPtg-db/db mice.

A novel and significant finding in the present study was that CRP mediated renal fibrosis via the CD32b-Smad3-mTOR signaling pathway. Increasing evidence shows that activation of TGF-β/Smad signaling contributes to accumulation of the extracellular matrix, resulting in progressive renal fibrosis in both animal and human diabetic kidneys^25^,^26^,^27^,^28^,^29^,^30^. We have previously shown that under diabetic and hypertensive disease conditions, AGEs and angiotensin II are capable of activating TGF-β/Smad3 signaling directly via the ERK/p38 MAP kinase-crosstalk mechanism and indirectly by inducing TGF-β^31^,^32^. The functional role for TGF-β/Smad3 in diabetic kidney disease comes from recent studies that deletion of Smad3 protects against diabetic kidney disease^29^,^33^. Results from the present study added a new finding that CRP was able to induce the early phosphorylation of Smad3 and ERK1/2 and p38 at 15 mins in tubular epithelial cells, which was blocked by a neutralizing CD32b antibody and inhibitors to the ERK1/2 or p38. These findings demonstrate that CRP induced an early Smad3 signaling via the CD32b-ERK/p38 MAP kinase crosstalk pathway. This was further confirmed by the inability of addition of a neutralizing TGF-β1 antibody to block the early activation of Smad3 in response to CRP. Because CRP alone is able to upregulate TGF-β1 production by renal tubular epithelial cells in a time- and dosage-dependent manner^9^, addition of a neutralizing TGF-β1 antibody was capable of inhibiting the late activation of Smad3 at 24 hrs after CRP stimulation, indicating a TGF-β1-dependent mechanism in CRP-induced late activation of Smad3 signaling. Thus, CRP bound CD32b to activate Smad3 via both TGF-β1-independent and dependent mechanism. This may account for the finding that activation of TGF-β1/Smad3 signaling and renal fibrosis was significantly enhanced in the diabetic kidney of CRPtg-db/db mice. Interestingly, TGF-β1 is capable of inhibiting IL-1β and IL-6-induced CRP production in a time and dosage-dependent manner^34^, suggesting a regulatory loop between CRP and TGF-β in renal fibrosis and inflammation. The interplay between CRP and TGF-β requires further investigation.

mTOR is a serine/threonine kinase that exists in two distinct signaling complexes known as mTOR Complex 1 (mTORC1) and mTORC2^35^. Emerging evidence shows that activation of mTOR plays a pivotal role in many kidney diseases including mesangial expansion, glomerular basement membrane thickening and tubulointerstitial fibrosis in diabetic nephropathy^14^,^15^,^36^,^37^,^38^,^39^,^40^,^41^. Multiple studies have shown that mTOR can be activated by TGF-β and contributes to the profibrotic effect on matrix protein production^16^,^42^. In the present study, we found that there was a Smad3 binding site on the UTR region of mTOR and Smad3 could physically bind to mTOR as demonstrated by a ChiP assay and western blot. CRP alone was able to enhance Smad3-mTOR interaction and induce mTOR/S6K activation and renal fibrosis. Blockade of the CRP signaling with an anti-CD32b antibody or Smad3 signaling with a specific inhibitor (SIS3) was able to inhibit mTOR signaling, thereby attenuating renal fibrosis including mesangial expansion under high CRP conditions *in vivo* and *in vitro*. Thus, activation of CD32b-Smad3-mTOR signaling may be a key mechanism by which CRP mediates renal fibrosis. Interestingly, the combination of CRP and high glucose did not additively enhance mTOR signaling, but this was blocked by a neutralizing antibody to CD32b, suggesting that high glucose may induce activation of mTOR via a CRP-dependent mechanism as high glucose is known to induce CRP^9^. The functional importance for the CD32b-Smad3-mTOR pathway in renal fibrosis was further demonstrated by the ability of rapamycin to inactivate the mTOR signaling, thereby inhibiting CRP and high glucose-induced CTGF and collagen I expression.

In conclusion, the present study demonstrates that CRP is pathogenic in type-2 diabetes and diabetic renal complication. CRP is capable of activating Smad3 signaling directly through the ERK/p38 MAP kinase-crosstalk pathway and indirectly via the TGF-β1-dependent mechanism. CRP may promote renal inflammation via the CD32b-NF-κB signaling mechanism, whereas, CRP may enhance renal fibrosis via the CD32b-Smad3-mTOR signaling pathway. Thus, results from this study suggest that targeting CRP may offer an alternative approach for prevention and treatment of diabetic kidney complication.

## Materials and Methods

### Animal model

Male db/db mice overexpressing human CRP (CRPtg-db/db) were used in this study. CRPtg-db/db mice were generated by crossbreeding db/m (C57BL/KSJ background) mice with human CRP transgenic mice (C57BL/6J background)^7^. Their littermates (CRPtg-db/m, WT-db/m and WT-db/db) were served as controls. The genotypes of mice were determined by genotyping of their tail DNA with specific CRP primers (forward, 5′-TTTACAGTGGGTGGGTCTGAAATA-3′ and reverse, 5′-GGGCCACAGCTGGGGTTTGGTGAA-3′) and db/db primers (forward, 5′-AGAACGGACACTCTTTGAAGTCTC-3′ and reverse, 5′-CATTCAAACCATAGTTTAGGTTTGTGT-3′). While the serum levels of human CRP levels were determined by serum ELISA (R&D System, Minneapolis, MN, USA). Groups of eight male mice were euthanized at week 36 and the renal cortexes were collected for histology, immunohistochemistry, western blotting, and real-time PCR analysis. Blood plasma was collected by cardiac puncture in anaesthetized mice. All studies were approval by the Animal Experimentation Ethics Committee, the Chinese University of Hong Kong and the experimental methods were carried out in accordance with the approved guidelines.

### Cell culture

The normal adult human TEC line, HK-2 cells, were cultured in serum-free DMEM (Dulbecco’s modified Eagle’s medium)/Ham’s F12 medium (Invitrogen Life Technologies, Gaithersburg, MD, USA) with recombinant human CRP (10 μg/ml; R&D Systems, Minneapolis, MN, USA) for various periods of time to detect the activation of Smad3 and mTOR. To block the CRP-CD32b interaction or ERK1/2 and p38 activities, HK-2 cells were pretreated with an anti-CD32b neutralizing antibody ((5 μg/ml; R&D), and specific inhibitors to ERK1/2 (PD98059, 20 μM; Biomol), to p38 (SB203580, 10 μM; Biomol) for 1 hour before addition of CRP (10 μg/ml) for 15 minutes. To block Smad3 activation, HK-2 cell was treated with or without SIS3 (10 μM, Sigma-Aldrich), a specific smad3 inhibitor for 1 hour before addition of CRP (10 μg/ml). To determine whether activation of mTOR induced by CRP was enhanced under high glucose conditions, HK-2 cells were cultured in FBS-free medium for 24 hours and then stimulated with CRP (10 μg/ml) under normal D-glucose (5.5 mM) or high D-glucose (35 mM) conditions for up to 6 hours, D-Mannitol (35 mmol/ml) was used as an osmotic control. To block CRP-mTOR signaling, HK-2 cells was treated with or without anti-CD32b neutralizing antibody (5 μg/ml; R&D) or mTOR inhibitor, rapamycin (10 μM; Sigma-Aldrich) for 1 hour before addition of CRP (10 μg/ml). All the experiments were repeated independently for at least three times.

### Fasting blood glucose

Blood glucose levels were measured by Accu-Chek glucose meter (Roche Diagnostics) after the mouse fasting for 6 hours as recommended by the Animal Models of Diabetic Complications Consortium^43^.

### Renal Function Measurement

Urine was collected by housing individual mouse in the metabolic cages for 24 hours. A competitive ELISA method was used to detect the urine micro-albumin according to the manufacturer’s instruction (Exocell, Philadelphia, PA, USA).

Levels of blood urea nitrogen (BUN) and serum or urinary creatinine were determined accordingly with enzymatic method (Stanbio Laboratories, TX, USA).

### Histopathology and Immunohistochemistry

Formalin-fixed, paraffin-sections (2–3 μm) were stained with Periodic Acid-Schiff’s (PAS) reagent and periodic acid-silver methenamine (PASM), Masson’s trichrome as previously described^44^. Immunohistochemistry was performed in paraffin sections by using a microwave-based antigen retrieval method^45^. The primary antibodies used in this study included as follows: TNF-α, IL-1β, TGF-β1 (Santa Cruz Biotechnology, Santa Cruz, CA), CD3 (eBioscience, San Diego, CA), F4/80 (AbD Serotec, Kidlington, UK), phospho-Smad3 (Rockland, Immuno-chemicals, Gilbertsville, PA), collagen I, collagen IV (Southern Biotech, Birmingham, AL), and Phospho- NF-κB/p65 (Abcam, Cambridge, MA, USA), Phospho-mTOR (Ser2448) (Cell Signaling Technology, Danvers, MA). Fcγ RIIB/CD32b (R&D). Sections were counterstained with hematoxylin for the nuclei. Positive signals were quantitatively analyzed using the quantitative Image Analysis System (Image-Pro plus 7.0, Media Cybernetics, Bethesda, MD, USA) as described previously^46^.

To examine the activation of p-mTOR induced by CRP, HK-2 cells were incubated with p-mTOR antibody (Cell Signaling) for overnight at 4 °C, followed by the goat anti-rabbit FITC-conjugated IgG (Zymed Laboratories, San Francisco, CA) for 1 hour. Nuclei were counterstained with DAPI. The p-mTOR positive cells were identified under fluorescent microscope (DM60008, Leica microsystems Ltd., Germany).

### Western blotting analysis

Protein from the kidney cortex or HK-2 cells was extracted and western blot analysis was performed as described previously^47^. Antibodies used in this study included: collagen I, collagen IV (Southern Biotech), CTGF, Phospho-p70s6k, p70s6k, β-actin (Santa Cruz), CD32b (R&D), Phospho- NF-κB/p65, NF-κB/p65, phospho-Smad3, Smad3, Phospho-mTOR (Ser2448), mTOR, Phospho-ERK1/2 MAPK, ERK1/2 MAPK, Phospho-p38 MAPK, p38 MAPK (Cell Signaling), and LI-COR IRDye 800-labelled secondary antibodies (Rockland Immuno-chemicals). The detection of specific signals was performed by using the Odyssey infrared image system (LI-COR Biosciences, Lincoln, NE, USA) and quantified by Image J software (National Institutes of Health). The ratio for the protein detected was normalized against β-actin and expressed as the mean ± S.E.M.

### RNA Extraction, Quantitative Real-Time PCR

Total RNA was extracted from the renal cortical tissues and cultured HK-2 cells and real-time PCR was performed with a real-time PCR machine (Option 2, Bio-Rad, Hercules, CA, USA) by using IQ SYBR green supermix reagent (Bio-Rad)^47^,^48^. The primers for mouse mRNA MCP-1, IL-1β, TNFa, collagen I, collagen IV, CD32 and human mRNA CTGF have been described previously^9^,^10^,^48^,^49^, The housekeeping genes β-actin was used as internal controls. The ratio of specific mRNA: β-actin mRNA was calculated using the 2^−ΔΔCt^ method and is expressed as the mean ± S.E.M.

### Chromatin immunoprecipitation analysis

Chromatin immunoprecipitation was performed as previously described^48^. Primary antibody against Smad3 (Cell Signaling) was used. Precipitated DNAs were detected by PCR using specific primers to detect the binding of Smad3 to mTOR: forward: 5′-GCAGCATCACTGGGTCTGAT-3′, reverse: 5′-GAAGATGCTGACCTCACCCC-3′.

### Flow cytometry

The HK-2 cells were treated with or without SIS3 (10 μM) for 1 hour before addition of CRP (10 μg/ml) for another 1 hour and examined for p-mTOR by flow cytometry. Briefly, the cells were fixed with 4% paraformaldehyde for 10 min and then permeabilized with 0.1% PBS-Tween 20 for 20 min and incubated with goat serum/PBS to block non-specific the anti-mTOR (phospho S2448) antibody (1:200; Abcam) at 4 °C overnight, followed by FITC-labeled goat anti-rabbit IgG (1:2000; Zymed). Isotype rabbit IgG was used as negative control. Acquisition of 10000 events was collected.

### Statistical analysis

All the data are expressed as the mean ± S.E.M. Statistical analyses were performed with one-way analysis of variance as appropriate, followed by Bonferroni’s post-hoc test. In addition, urine albumin excretion and fasting blood glucose levels were assessed by using a two-way analysis of variance. Tests were performed with GraphPad Prism 5 (GraphPad Soſtware, La Jolla, CA). P-value < 0.05 was considered significant.

## Additional Information

**How to cite this article**: You, Y.-K. *et al.* C-Reactive Protein Promotes Diabetic Kidney Disease in db/db Mice via the CD32b-Smad3-mTOR signaling Pathway. *Sci. Rep.* **6**, 26740; doi: 10.1038/srep26740 (2016).

## Supplementary Material

Supplementary Information
