# Supplementary material for: C-Reactive Protein Promotes Diabetic Kidney Disease in db/db Mice via the CD32b-Smad3-mTOR signaling Pathway
Source: Sci Rep. 2016 May 25;6:26740. doi: 10.1038/srep26740 (PMC4879671; doi:10.1038/srep26740)
Supplement: Supplementary Information [file srep26740-s1.pdf]

Supplementary Information

C-Reactive Protein Promotes Diabetic Kidney Disease in db/db Mice via the CD32b-Smad3-mTOR signaling Pathway

Yong-Ke You [1, 2] Xiao-Ru. Huang[2], Hai-Yong Chen[2], Xia-Fei Lyu[2], Hua-Feng Liu[1]*, Hui Y. LAN[2]*

[1]Institute of Nephrology, Guangdong Medical College, Zhanjiang, Guangdong, and

[2]Department of Medicine and Therapeutics, and Li Ka Shing Institute of Health Sciences, and Shenzhen Research Institute, the Chinese University of Hong Kong, Hong Kong, China;

Running Title: Role of CRP in T2DN.

* Co-corresponding authors

Address correspondence to:

Prof. Hui Yao Lan, Department of Medicine and Therapeutics, and Li Ka Shing Institute of Health Sciences, The Chinese University of Hong Kong, Prince of Wales Hospital, Shatin, New Territories, Hong Kong, China, Tel: +852-37636077, Fax: +852-21457190, E-mail: hylan@cuhk.edu.hk; and Professor Hua-Feng Liu, Institute of Nephrology, Guangdong Medical College, Zhanjiang, Guangdong, China, E-mail: hf-liu@263.net.

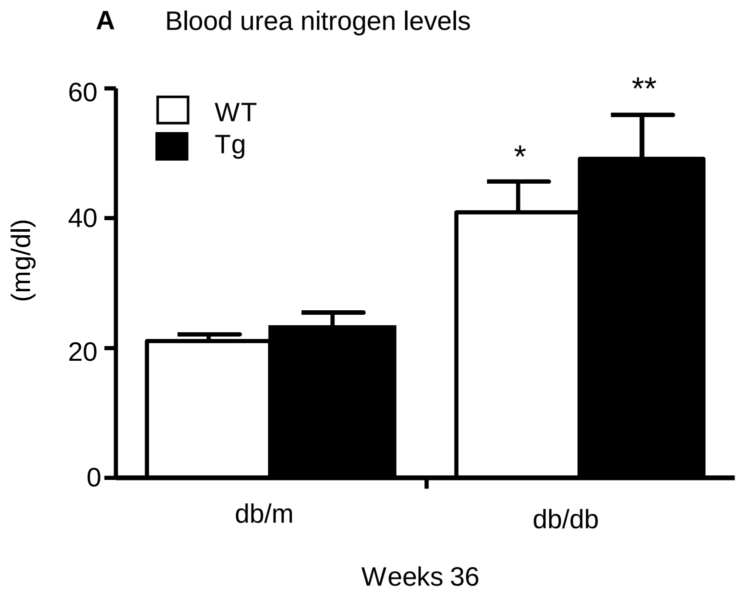

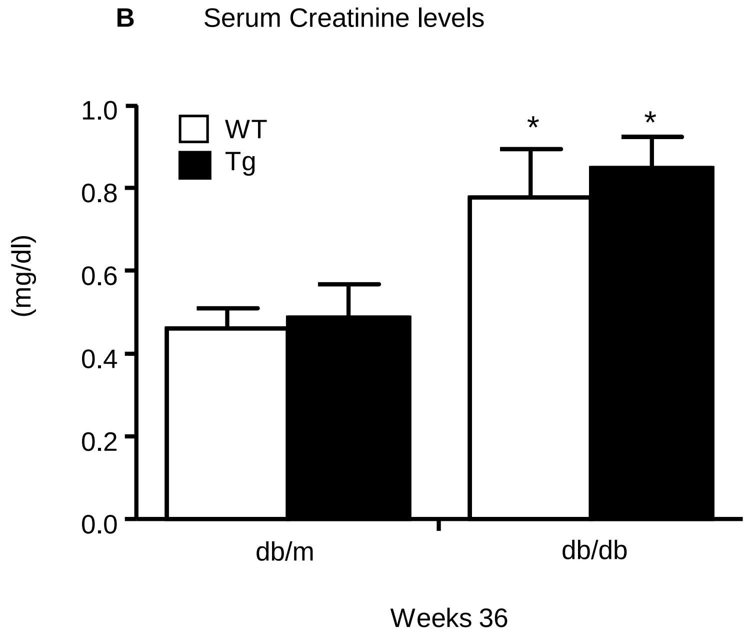

Figure S1. Levels of blood urea nitrogen (BUN) and serum creatinine. **(A)** BUN and **(B)** serum creatinine. The levels of blood urea nitrogen (BUN) and serum creatinine are only marginally increased in CRPtg-db/db mice. Data represents the mean ± SEM for eight mice per group. *p<0.05, **p<0.01 compared with db/m mice.

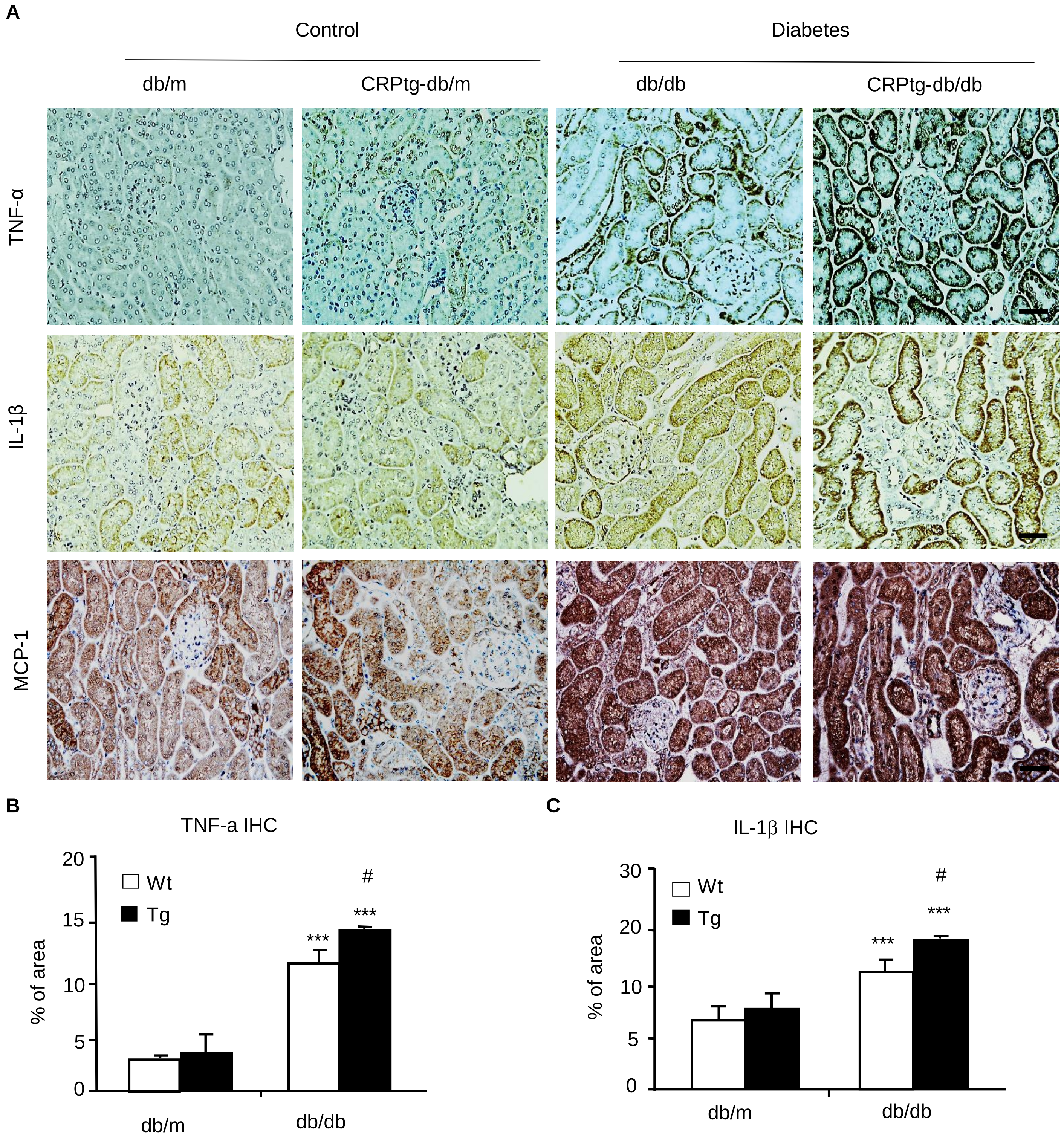

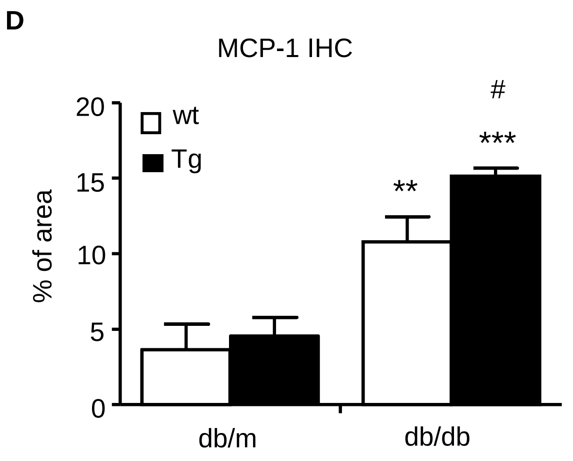

Figure S2. CRPtg-db/db mice develop more severe renal inflammation with a marked upregulation of pro-inflammatory cytokines.

(A) Immunohistochemical staining. **(B-D)** Quantitative analysis of TNF-α, IL-1β and MCP-1. Data represents the mean ± SEM for eight mice per group. Bar=50μm. ***p*<0.01, ****p*<0.001 compared with db/m mice; #*p*<0.05 compared with db/db mice.

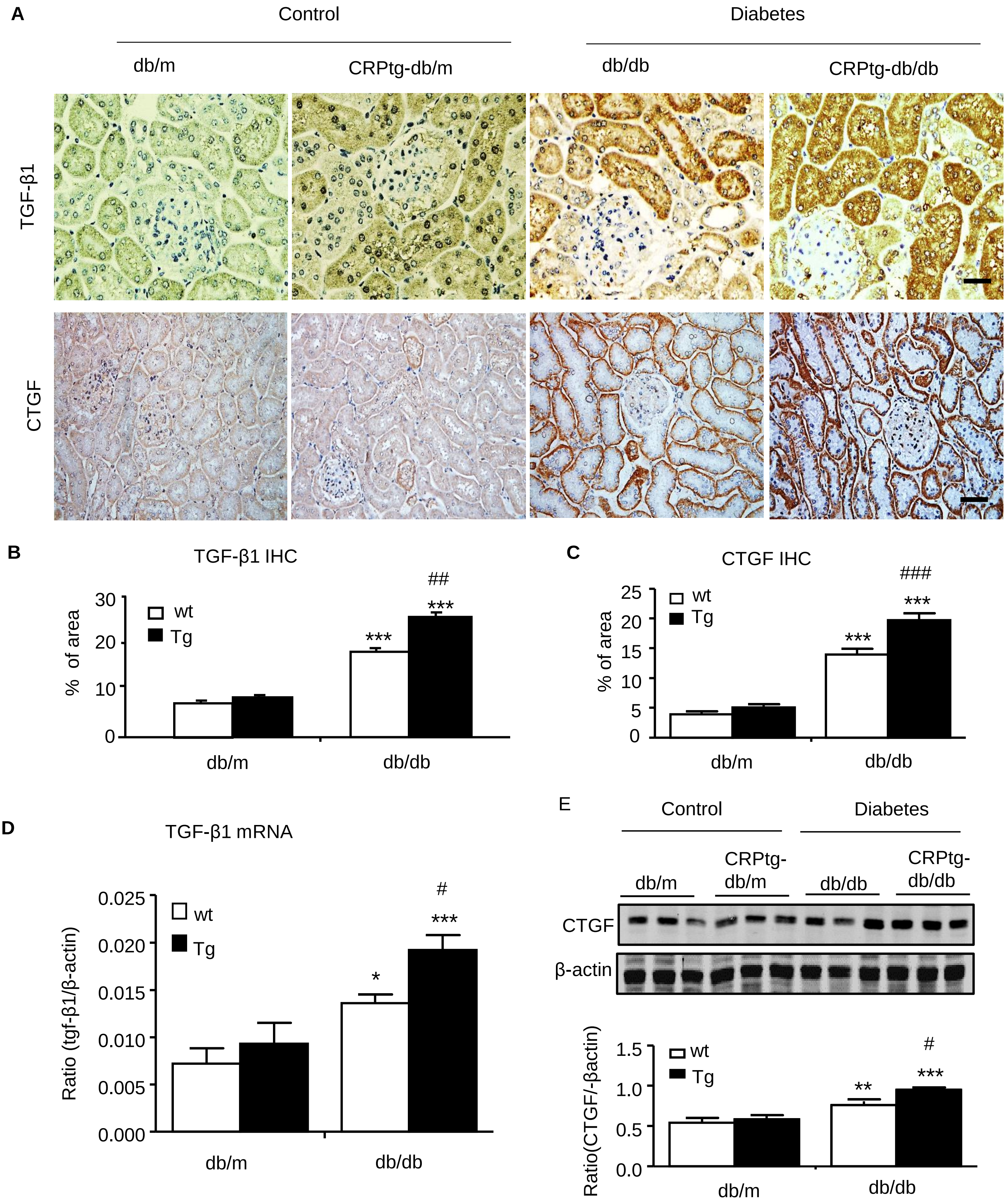

Figure S3. Expression of TGF-β1 and CTGF is markedly upregulated in CRPtg-db/db mice. (A-C) Immunohistochemical staining and quantitative analysis of TGF-β1 and CTGF. **(D)** Real-time PCR analysis of TGF-β1 mRNA expression. **(E)** Western blot analysis of CTGF. Data represents the mean ± SEM for eight mice per group. Bar=50µm. *p<0.05, **p<0.01, ***p<0.001 compared with db/m mice; #p<0.05, ##p<0.01, ###p<0.001 compared with db/db mice.

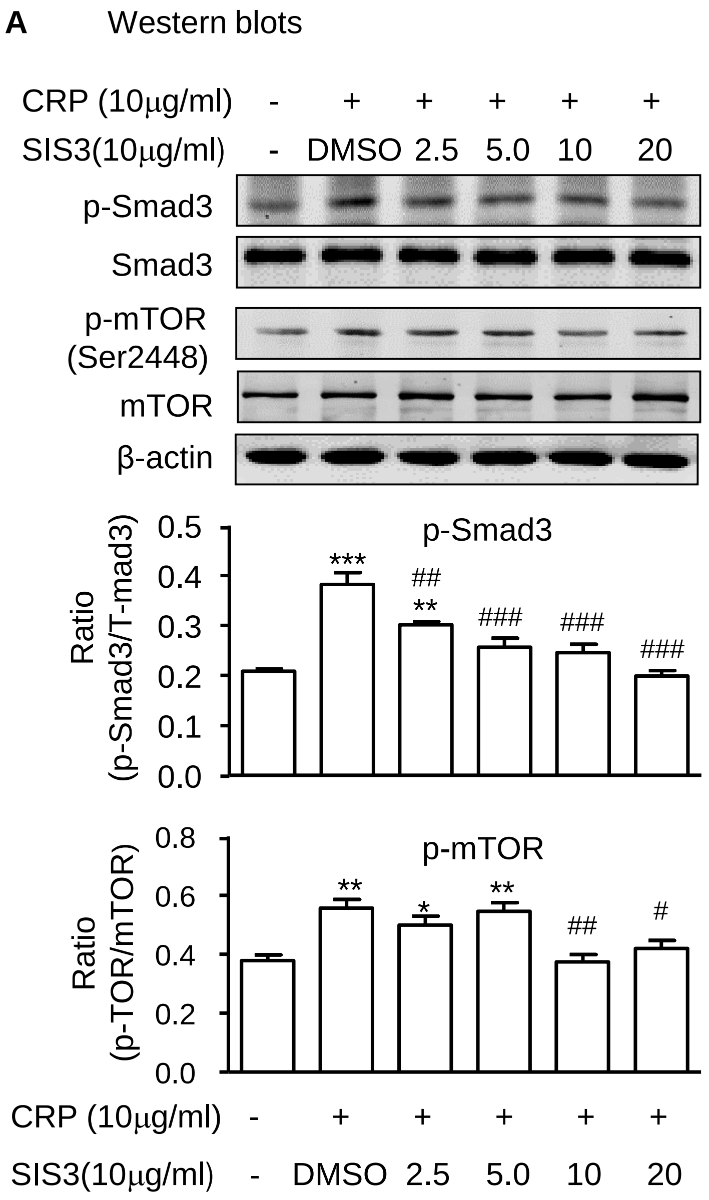

Figure S4. Blockade of Smad3 signaling with SIS3 was able to inhibit CRP-induced mTOR signaling in HK-2 cells. (A) HK-2 cells were pre-treated with SIS3 at different concentrations (2.5, 5, 10, and 20μM) or DMSO for 1 hour before stimulated with CRP (10μg/ml) for 1 hour. Representative western blots and quantitative analysis of p-Smad3 and p-mTOR are shown. Data represents the mean ± SEM for at least three independent experiments. *p<0.05, **p<0.01, ***p<0.001 compared with normal group; #p<0.05, ##p<0.05, ###p<0.05 compared with addition of CRP.

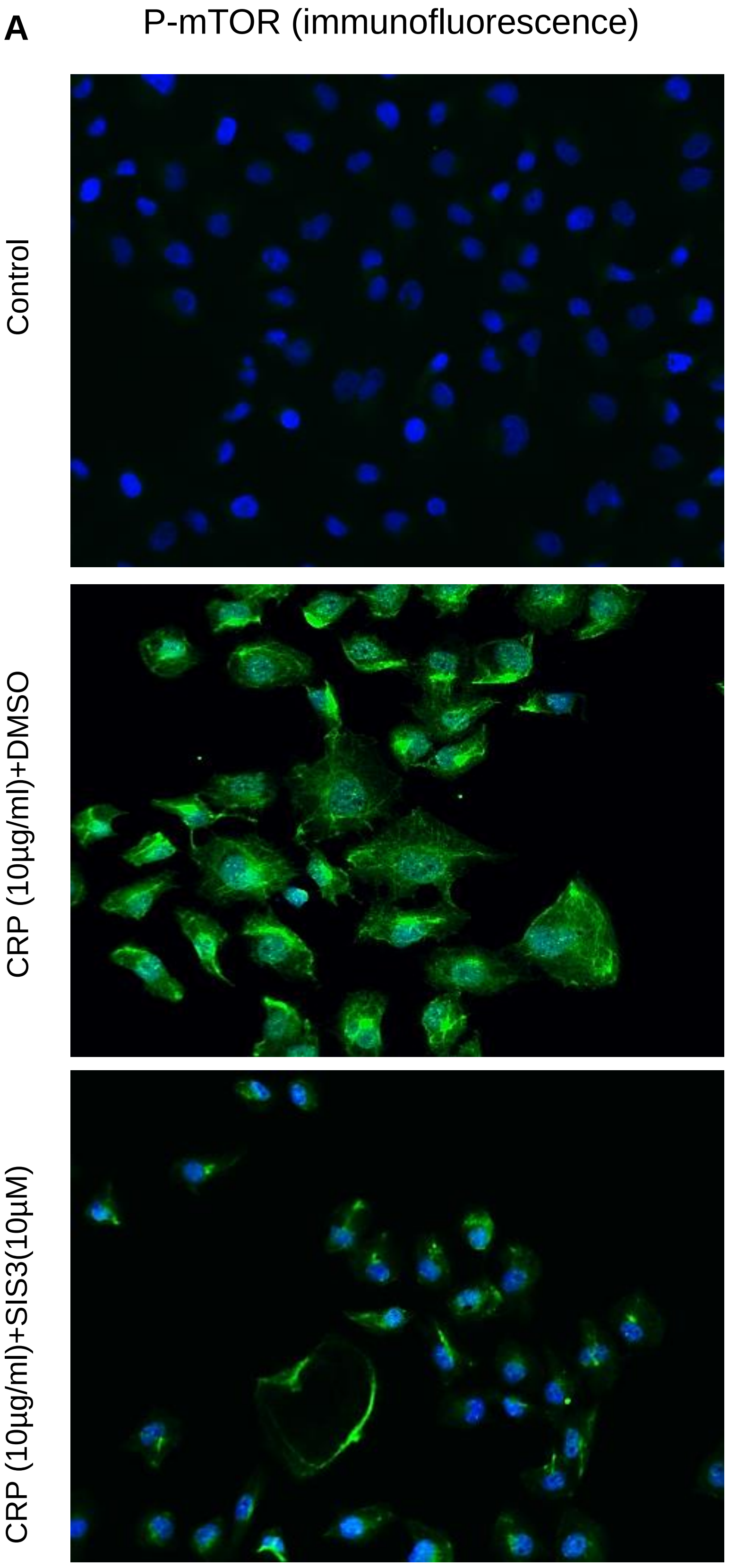

Figure S5. Blockade of Smad3 signaling with a specific inhibitor (SIS3) was able to inhibit CRP-induced phosphorylation of mTOR in HK-2 cells.
